# Factors associated with malnutrition in hospitalized cancer patients: a croos-sectional study

**DOI:** 10.1186/s12937-015-0113-1

**Published:** 2015-12-10

**Authors:** Fernanda Rafaella de Melo Silva, Mirella Gondim Ozias Aquino de Oliveira, Alex Sandro Rolland Souza, José Natal Figueroa, Carmina Silva Santos

**Affiliations:** Institute of Integrative Medicine Prof. Fernando Figueira (IMIP), Recife, PE Brazil; Federal University of Pernambuco (UFPE), Recife, PE Brazil

**Keywords:** Nutrition assessment, Risk factors, Malnutrition, Cancer

## Abstract

**Introduction:**

The incidence of cancer is increasing worldwide and with it the prevalence of malnutrition, which is responsible for the death of almost 20 % of cancer patients. The objective of this study was to identify the factors associated with malnutrition in hospitalized cancer patients.

**Methods:**

Cross-sectional study conducted with 277 hospitalized patients in the Institute of Integrative Medicine Prof. Fernando Figueira from March to November 2013. The nutritional status was classified as well-nourished and moderate/severe malnutrition, according to the Patient-Generated Subjective Global Assessment. The association between moderate/severe malnutrition and demographic, behavioral, socioeconomic, clinical, therapeutic and nutritional variables was investigated through univariate regression and hierarchical Poisson models, with a 5 % significance level.

**Results:**

The prevalence of malnutrition was 71.1 %, being classified as moderate in 35.4 % and severe in 35.7 %. After multivariate analysis, smokers/ex-smokers low socioeconomic status, performance status ≥2 and age ≥60 years were associated with increased risk of malnutrition.

**Conclusion:**

There was observed a high prevalence of moderate/severe malnutrition in cancer patients, with the increased risk of malnutrition associated with the presence of factors that can be assessed during hospital admission suggesting a higher alert of the medical and health care staff about the need for nutritional assessment and intervention.

## Introduction

The incidence of cancer is increasing worldwide and with it the prevalence of malnutrition, which may vary between 40 and 80 % in patients with neoplasia [1, 2]. The cancer is responsible for approximately 13 % of all causes of death worldwide, however the death of almost 20 % of cancer patients occurs as a result of malnutrition and its complications, rather than the malignancy of the disease itself [3, 4].

In Brazil, a multicenter study found a prevalence of malnutrition of 66.3 % and an increased risk of almost three-fold of malnutrition (20.3 %) among cancer patients [5]. A similar result was found in a study conducted in Latin America with hospitalized patients, which showed a prevalence of 65.6 % of malnutrition in individuals with cancer [6].

The etiology of malnutrition in cancer patients is complex and multifactorial and may be influenced by the location and type of tumor, stage of the disease, side effects of the treatment, socioeconomic status, functional performance, symptoms of nutritional impact, need for fasting and inadequate nutritional therapy, as well as medical staff awareness about the importance of nutritional status for the prognosis and quality of life of hospitalized patients [5–8].

In patients with cancer, a risk up to 30 % of malnutrition among hospitalized patients in older age (OR 1.38; IC 95 %: 1.28 – 1.54) [6] and almost three-fold in patients with low functional performance [3] were observed. Another study found that smoking is associated with the need for enteral nutrition therapy in patients with head and neck cancer, suggesting that is also associated with depletion of the nutritional status, because this therapy is used when there is a partial or total impairment of food intake. However, these factors are not well elucidated [9].

The identification of factors that can be assessed during the hospital admission can alert the medical and healthcare staff about the need for evaluation and early nutritional intervention to avoid further complications. Researches show that malnutrition is associated with lower response and tolerance to antineoplastic treatment; reduction in the quality of life; higher morbidity, mortality and infectious complications and an increase in length of hospital stay and costs by reducing the availability of hospital beds and consequently the number of patients attended [3, 10].

The objective of this study was to determine the prevalence and factors associated with malnutrition in hospitalized cancer patients so they can be identified at hospital admission and possible complications from malnutrition avoided.

## Methods

Cross-sectional study, performed between March and November 2013, in oncology and palliative care inpatient units of the Institute of Integrative Medicine Prof. Fernando Figueira (IMIP), referral center for oncology in northeastern Brazil, located in Recife, Pernambuco. The study started after its approval by the Ethics Committee on Human Research of the cited institution under protocol 10241612.7.0000.5201.

Patients with cancer of both sexes and aged 18 and older participated in the study. Those patients hospitalized for more than 72 h, who where fed exclusively through catheter or ostomies for more than 72 h, with consumptive disorders or pregnant were excluded from the sample.

The sample size was calculated using the Statcalc function of the Epi-Info 3.5.3 program (*Centers for Disease Control and Prevetion*, Atlanta, GA, EUA). Considering a frequency of malnutrition in cancer patients of 30 % [3], a significance level of 5 %, an 80 % power and a relative accuracy of 20 %, would be necessary 224 individuals. Foreseeing possible losses, the sample was increased by 20 % to 277 volunteers.

The selection and evaluation of the participants were performed by a single trained nutritionist. After the sign of an Informed Consent Form, by the patient or legal guardian, were collected demographic, behavioral and socioeconomic variables [age (years), gender, origin, marital status, occupation, smoking, education, and socioeconomic class]; nutritional variables [symptoms of nutritional impact, current weight (kg), habitual weight (kg), height (m), weight loss percentage and previous body mass index (kg/m^2^)]; clinical variables (type of cancer, presence of metastasis and performance status) and therapeutic variables (previous treatment and type of anticancer treatment).

Demographic, behavioral and socioeconomic variables were collected according to the report of the patient and medical record and socioeconomic class and education were obtained from the Brazilian Association of Research Companies questionnaire (ABEP) [11]. According to the ABEP questionnaire, family income is classified in five levels, being A the highest and E the lowest socioeconomic level. For analysis purposes, the extremes of socioeconomic levels were grouped to form three categories: A and B; C; D and E [11].

To obtain the anthropometric variables (current weight and height) the patient was measured twice and if there was difference between these values a third measure was made and then calculated the average and recorded the final value [12]. To measure these variables it was used a fixed vertical digital platform scale with stadiometer (FILIZOLA™, São Paulo, Brazil) with a variation of 50 g, capacity up to 200 kg and 0.5 cm precision. If there was no possibility of measuring the stature, it was estimated from the Chumlea equation [13]. Habitual weight (HW) of the last six months was obtained according to the report of the patient.

The weight loss percentage (%WL) was calculated from the equation [(habitual weight - current weight)/habitual weight × 100], considering the cutoff point of %WL ≥5 on the past six months [14–16]. The previous body mass index (pBMI) was obtained by the equation (habitual weight/height^2^) and was classified according to the recommendations of the World Health Organization (WHO) for adults and the Pan American Health Organization (PAHO)/WHO for elderly [12, 17]. This variable was stratified into two categories corresponding to overweight (overweight and obese) and normal weight (normal weight and malnutrition).

Nutritional diagnosis was obtained through the scored Patient-Generated Subjective Global Assessment (PG-SGA), which consists of a tool used for screening and nutritional diagnosis of cancer patients developed for Ottery [14] and validated in Portuguese by Gonzalez et al. [15]. This questionnaire allows not only classify the nutritional assessment into three categories (A = well nourished, B = suspected or moderate malnutrition and C = severe malnutrition), but also generates a numerical score which enables the selection of the appropriate level of nutritional intervention [14, 15]. For statistical analysis, the participants who were in categories B and C were classified as malnourished nutritional status. The presence of symptoms of nutritional impact was also obtained from this questionnaire.

Clinical variables were collected in medical records, but the performance status (PS) was obtained from the scale developed by the *Eastern Cooperative Oncology Group* (ECOG) [18], that ranks the functional capacity in five levels, being the zero, the fully active individual and the four, the bedridden and unable to perform self-care activities individual. Considering that the limited functional capacity leads to difficulties in preparing and food intake, this variable was stratified in PS <2 and PS ≥2, because PS = 2 indicates inability to perform any work activity [19, 20].

For analysis, the cancers were stratified into six groups according to the location of the tumor (upper gastrointestinal tract, lower gastrointestinal tract, reproductive tract, lung, breast and other cancers). The cancers belonging to the latter class were those who do not fit into any of the former classifications.

Regarding therapeutic variables, the types of treatment were stratified into clinical treatment, when the individual has been subjected to at least one radiotherapy and/or chemotherapy session; surgical treatment, when any procedure for complete or partial resection of the tumor mass was conducted; medical/surgical treatment, when both treatments were needed; and palliative treatment when the therapy performed had no more intention of healing.

In order to avoid bias, the evaluations were performed by a single trained examiner and the questions asked in the same way for all studied individuals. The PG-SGA was preferably the first evaluation to be conducted. Before the anthropometric measurements the calibration of the instruments was always checked.

Categorical variables were presented by absolute and relative frequencies and the continuous variables were summarized using the central tendency and dispersion measures. The association between malnutrition and analytical variables was performed by the Poisson regression model, assuming a significance level of *p* <0.05. To adjust the effect of independent predictors for malnutrition, all variables were included in a new hierarchical Poisson regression model, adopting a significance level of 5 %, a 95 % confidence interval and a power of 80 %. Data analyses were conducted through STATA 12.1SE program (*Stata Corporation*, *College Station*, TX, USA).

## Results

During the study period 480 cancer patients were admitted, being 203 ineligible by exclusion criteria. Thus, 277 patients were eligible and all agreed to participate.

More than half of the sample (56.0 %) were female. The mean age was 54.7 ± 14.9 years, with 39.4 % (*n* = 109) of patients aged ≥60 years. Most individuals (60.3 %) had a partner and 37.9 % came from the countryside of Pernambuco. In relation to scholarity, 40.1 % had not finished elementary school. The results showed that most of the study population belonged to the lower social classes: Class C (52.7 %); and classes D and E (23.8 %). Most (61.0 %) were retired or had illness benefit and 14.1 % worked (Table 1).

**Table 1:** Sociodemographic characteristics of the sample

Characteristics	N(%)
Marital status	
With partner	167 (60.3)
Without partner	110 (39.7)
Origin	
Recife	96 (34.7)
RMA^a^	69 (24.9)
Countryside	105 (37.9)
Another states	7 (2.5)
Scholarity	
Unlettered	40 (14.4)
Incompleted elementary school	111 (40.1)
Completed elementary school	28 (10.1)
Completed high school	82 (29.6)
Completed undergraduation	16 (5.8)
Socioeconomic class	
A and B	65 (23.5)
C	146 (52.7)
D and E	66 (23.8)
Ocupation	
Paid work	39 (14.1)
Retired/Benefit	169 (61.0)
Unemployed	44 (15.9)
Others	25 (9.0)
Smoking	
Smoker/ex-smoker	138 (49.8)
Never smoke	139 (50.2)

^a^Recife metropolitan area

The types of cancers, classified by the organic system, most frequently affected were the lower gastrointestinal tract (LGIT) (20.9 %) and reproductive system (18.8 %). Although the majority of patients (62.1 %) had received any type of curative treatment, half of them (50.2 %) had metastatic tumors. The sample showed a considerable prevalence of impaired physical performance (46.2 %), being more prevalent in tumors of the reproductive system, as well as the symptoms of nutritional impact (Table 2).

**Table 2:** Sample characteristics according to the types of cancer

		Type of cancer					
Characteristics	Total sample	UGIT^a^	LGIT^b^	RS^c^	Lung	Breast	Others^d^
Type of cancer - n (%)	277 (100)	43 (15.5)	58 (20.9)	52 (18.8)	32 (11.6)	30 (10.8)	62 (22.4)
Age - Mean (SD)	54.7 (14.9)	57.6(13.3)	57.6 (12.9)	52.9 (15.3)	57.7 (13.8)	56.6 (15.5)	48.8 (16.2)
≥60 - n (%)	109 (39.4)	20 (18.3)	29 (26.6)	19 (17.4)	13 (11.9)	12 (11.0)	16 (14.7)
Gender - n (%)							
Male	122 (44.0)	23 (18.9)	20 (16.4)	15 (12.3)	21 (17.2)	0 (0.0)	43 (35.2)
Female	155 (56.0)	20 (12.9)	38 (24.5)	37 (23.9)	11 (7.1)	30 (19.4)	19 (12.3)
Metastasis - n (%)							
Absent	138 (49.8)	15 (10.9)	29 (21.0)	26 (18.8)	11 (8.0)	19 (13.8)	38 (27.5)
Present	139 (50.2)	28 (20.1)	29 (20.9)	26 (18.7)	21 (15.1)	11 (7.9)	24 (17.3)
Performance status - n (%)							
<2	149 (53.8)	20 (13.4)	36 (24.2)	23 (15.4)	14 (9.4)	17 (11.4)	39 (26.2)
≥2	128 (46.2)	23 (18.0)	22 (17.2)	29 (22.7)	18 (14.1)	13 (10.2)	23 (18.0)
Symptoms of impact - n (%)							
Yes	186 (67.1)	31 (16.7)	35 (18.8)	39 (21.0)	26 (14.0)	17 (9.1)	38 (20.4)
No	91 (32.9)	12 (13.2)	23 (25.3)	13 (14.3)	6 (6.6)	13 (14.3)	24 (26.4)
%WL ≥5 em 6 meses - n (%)							
Yes	101 (80.2)	20 (19.8)	21 (20.8)	15 (14.9)	15 (14.9)	5 (5.0)	25 (24.8)
No	25 (19.8)	2 (8.0)	5 (20.0)	7 (28.0)	1 (4.0)	4 (16.0)	6 (24.0)
Overweight (pBMI) - n (%)							
Yes	76 (60.3)	14 (18.4)	12 (15.8)	15 (19.7)	12 (15.8)	4 (5.3)	19 (25.0)
No	50 (39.7)	8 (16.0)	14 (28.0)	7 (14.0)	4 (8.0)	5 (10.0)	12 (24.0)
PG-SGA - n (%)							
Well nourished	80 (28.9)	7 (8.8)	19 (23.8)	13 (16.3)	5 (6.3)	13 (16.3)	23 (28.8)
Moderate malnutrition	98 (35.4)	8 (8.2)	21 (21.4)	21 (21.4)	17 (17.3)	12 (12.2)	19 (19.4)
Severe malnutrition	99 (35.7)	28 (28.3)	18 (18.2)	18 (18.2)	10 (10.1)	5 (5.1)	20 (20.2)

*SD* standard deviation, *PS* performance status, *%WL* weight loss percentage, *PG-SGA* patient-generated subjective global assessment; ^a^Upper gastrointestinal tract: esophagus, stomach, pancreas, liver, gallbladder, biliary ducts; ^b^Lower gastrointestinal tract: colon, rectum and anal canal; ^c^Reproductive system: ovarian, cervical, testicular and prostate; ^d^Head and neck, hematological, unknown primary site, sarcoma, melanoma

The prevalence of malnutrition was 71.1 %, with similar frequencies between moderate (35.4 %) and severe malnutrition (35.7 %). In relation to the types of cancer, severe malnutrition was more prevalent in patients with cancer of the upper gastrointestinal tract (28.3 %). Of the 126 patients who remembered the usual weight, 80.2 % had %WL ≥5 in the last 6 months with a median of 14.7, whereas in tumors classified as others, 24.8 % had WL% ≥5 in the last 6 months (Table 2).

Univariate analysis revealed that smokers/ex-smokers, socio-economic classes C, D and E, tumors of the upper gastrointestinal tract and lung, patients who had not cancer treatment, palliative care, PS ≥2, individuals without a prior excess of weight and age ≥60 years were associated with the presence of malnutrition (Table 3).

**Table 3:** Unadjusted analysis of factors associated with malnutrition

Variables	Total sample	Malnutrition	PR (IC95%)	*p* value
	N	N (%)		
Smoking				0.005
Smoker/ex-smoker	138	109 (79.0)	1.25 (1.07 – 1.45)	
Never smoke	139	88 (63.3)	1.0	
Socioeconomic class				0.003
A and B	65	36 (55.4)	1.0	
C	146	106 (72.6)	1.31 (1.03 – 1.67)	
D and E	66	55 (83.3)	1.50 (1.18 – 1.92)	
Type of cancer				0.026
UGIT^a^	43	36 (83.7)	1.48 (1.05 – 2.08)	
LGIT^b^	58	39 (67.2)	1.19 (0.83 – 1.70)	
RS^c^	52	39 (75.0)	1.32 (0.93 – 1.88)	
Lung	32	27 (84.4)	1.49 (1.05 – 2.11)	
Breast	30	17 (56.7)	1.0	
Others^d^	62	39 (62.9)	1.11 (0.77 – 1.60)	
Previous treatment				0.005
Absent	60	50 (83.3)	1.23 (1.06 – 1.42)	
Present	217	147 (67.7)	1.0	
Type of treatment				<0.001
Clinical^e^	85	50 (58.8)	0.86 (0.62 – 1.21)	
Surgical	22	15 (68.2)	1.0	
Clinical e surgical	65	39 (60.0)	0.88 (0.62 – 1.25)	
Palliative	45	43 (95.6)	1.40 (1.05 – 1.88)	
Performance status				<0.001
<2	149	84 (56.4)	1.0	
≥2	128	113 (88.3)	1.57 (1.34 – 1.83)	
Overweight				0.023
Yes	76	64 (84.2)	1.0	
No	50	48 (96.0)	1.14 (1.02 – 1.28)	
Age				<0.001
<60	168	102 (60.7)	1.0	
≥60	109	95 (87.2)	1.44 (1.25 – 1.65)	

*PR* prevalence ratio, *CI* confidence interval, *p* significance level; ^a^Upper gastrointestinal tract: esophagus, stomach, pancreas, liver, gallbladder, biliary ducts; ^b^Lower gastrointestinal tract: colon, rectum and anal canal; ^c^Reproductive system: ovarian, cervical, testicular and prostate; ^d^Head and neck, hematological, unknown primary site, sarcoma, melanoma; ^e^radiotherapy and/or quimiotherapy

In multivariate analysis the factors that remained significantly associated with malnutrition were: smokers/ex-smokers, socio-economic classes C, D and E, PS ≥2 and age ≥60 years (Table 4).

**Table 4:** Independent factors associated with malnutrition

	Sample	Outcome				
Variables	N	N(%)	RR_unadjusted_(CI95%)	*p* value	RR_adjusted_(CI95%)	*p* value
*Distal level*						
Smoking				0.005		0.015
Smoker/ex-smoker	138	109(79.0)	1.25(1.07 – 1.45)		1.21(1.04 – 1.40)	
Never smoke	139	88(63.3)	1.0		1.0	
Socioeconomic class				0.003		0.012
A and B	65	36(55.4)	1.0		1.0	
C	146	106(72.6)	1.31(1.03 – 1.67)		1.30(1.02 – 1.65)	
D and E	66	55(83.3)	1.50(1.18 – 1.92)		1.44(1.13 – 1.84)	
*Intermediate level*						
Performance status				<0.001		<0.001
<2	149	84(56.4)	1.0		1.0	
≥2	128	113(88.3)	1.57(1.34 – 1.83)		1.50(1.30 – 1.75)	
*Proximal level*						
Age				<0.001		<0.001
<60	168	102(60.7)	1.0		1.0	
≥60	109	95(87.2)	1.44(1.25 – 1.65)		1.36(1.19 – 1.56)	

*RR* risk ratio, *CI* confidence interval, *p* significance level

## Discussion

In this study, according to PG-SGA, more than half of the sample had some degree of malnutrition, being similar to the frequency of moderate and severe malnutrition, 35.4 and 35.7 %, respectively. The factors that independently associated to this nutritional disorder were smokers/ex-smokers, socio-economic classes C, D and E, PS ≥2 and age ≥60 years.

The high prevalence of malnutrition found in our study is not surprising, considering that hospitalized cancer patients are more likely to have some level of nutritional disability compared to other hospitalized individuals [5, 6]. We must consider that the hospitalization prior to the period of the study was not an exclusion criterion in our research and this may have influenced the high prevalence. However, our results were similar to two Brazilian cross-sectional studies, which found a malnutrition prevalence of 66.3 and 77.8 %, although they used for nutritional diagnosis the Subjective Global Assessment (SGA) [5, 21] different from our study that used the PG-SGA. Equivalent results were also obtained in Latin America (65.6 %), South Korea (61.3 %) and Australia (76.0 %) [2, 6, 22].

In relation to lower prevalences of malnutrition in cancer patients, were found two other Brazilian studies, with frequencies of 39.4 and 48.2 %, using the PG-SGA as a nutritional assessment method. These results can be justified because they are studies conducted with patients at the beginning of the chemotherapy treatment and with more than 1/3 of the sample with tumor in stages I or II. Different from our patients who had 50.2 % of metastatic tumors, indicating advanced stages of disease [15, 23].

The most common method for initial nutritional assessment in cancer patients was the ASG [5, 21], however, nowadays this tool is not well recommended. Because the incorporation of prognostic indicators such as the details of the symptoms of nutritional impact and weight loss, which are frequently observed in patients with cancer, to the PG-SGA, this tool is the indicated as initial method of nutritional assessment on admission of these individuals [9, 14, 16, 24].

It is well known that the %WL >5 in 6 months previous to the diagnosis is correlated to a lower survival rate and when it is >10 % is associated with an increased risk of complications due to malnutrition [14]. In our study 36.5 % of the total sample had %WL≥5 in 6 months. This fact is a concern, because the weight loss process, regardless of the usual weight of the individual, is considered itself a malnutrition process, even after the body changes, the patient remains within normal patterns [12, 25].

Another characteristic prognostic indicator of the cancer patient is the presence of symptoms of nutritional impact, due to its high prevalence mainly due to the tumor itself, antineoplastic therapy introduced as well as malnutrition [14, 15]. Our results showed that more than half of the sample (67.1 %) had symptoms of nutritional impact, in which the most prevalent were: anorexia (50.5 %), pain (23.1 %), vomiting (19.4 %), constipation, and dysgeusia (17.7 %). These results agree with Brazilian and American cross-sectional studies, which place anorexia and pain among the most prevalent symptoms in their populations [22, 23].

A recent prospective study conducted in Canada on patients with advanced cancer showed an association between the symptoms of nutritional impact (anorexia, gastric fullness, dysgeusia, dry mouth and dysphage) and lower survival rate on its univariate analysis, with dysphagia as an independent factor on its final model [26]. This fact highlights the importance of evaluation and appropriate management of these symptoms, given the large impact that causes on prognosis and quality of life of patients.

A concerning data found was the high prevalence of severe malnutrition (35.7 %), because this value is above the presented in the literature [3, 15, 23, 27]. We could refer the high rate to the fact that our patients were exposed to various risk factors for malnutrition as observed in the univariate analysis. We must also highlight the fact that the food and nutrition insecurity can permeate many subjects of our population, since a part of it belongs to lower social classes, which is an independent risk factor for malnutrition (*p* = 0.012) in our multivariate analysis.

The data regarding nutritional status of our patients only reaffirm the neglect to recognize malnutrition as a public health problem, considering that the prevalence rates have not decreased, despite being well documented over the past decades and its relationship with the prognosis and quality of life of patients with cancer [1]. A limiting factor in our study for this assessment is the lack of data on early nutritional support for these patients. Although, an important move to be made is to invest in adequate nutritional screening in order to establish an early nutritional therapy in order to prevent deterioration of nutritional status which is already expected in hospitalized patients.

In this study, smoking was identified as an independent risk factor for malnutrition (*p* = 0.015), in agreement with a British retrospective study that found an association between the use of cigarettes above 20 units/day and the necessity of use of enteral nutrition in patients with head and neck cancer (OR 4.08; IC 95 %: 1.29 – 12.89) [9]. Among the deleterious effects of nicotine, its action on the central nervous system leads to reduced appetite and these individuals are also susceptible to taste disturbance, causing a lower intake of nutrients [9, 28]. In addition, the tobacco has a thermogenic effect, leading to weight loss [9]. Another important factor in this relationship between smoking and malnutrition is the economic impact of the cigarettes on family spent, because instead of buying groceries part of the money is spent to keep the addiction [29].

Functional autonomy is a factor that deserves attention from health professionals when the point is nutritional risk indicators, considering that individuals with limited functional capacity have difficulties in the preparation and food intake [19, 30]. The results of this study support this statement, because it was observed that the PS ≥2 was a risk factor for malnutrition. Similar results were found by a French epidemiological study that found a chance of being malnourished almost three-fold higher in cancer patients admitted with low functional performance [3]. Similarly a multicenter cohort study showed that this is also a risk factor for ambulatory patients [19].

A Greek prospective study conducted with 173 patients with lung cancer showed an association between the PS and the %WL >5 in the last 3 months (*p* <0.001), with %WL as an indicative of a considerable deterioration of the nutritional status [14, 16, 27]. Other results have also shown an independent relationship of the performance status with survival and death of patients with cancer [3, 26, 31].

According to our results, elderly is a factor that increases by almost 30 % the chance of malnutrition. This association was expected, since it is well known that advanced age predisposes to nutritional deficiencies, especially in hospitalized individuals [3, 5, 6].

The previous overweigh was not associated with malnutrition in this study, most likely due to the limited number of patients (*n* = 126) who remembered the usual weight in the last 6 months. A French research showed a significant association between prior obesity and risk of malnutrition [3]. This finding can be explained by the negligence in relation to the excess of weight loss by the health care team, even knowing that the decrease in weight can be of lean mass, resulting in a worse prognosis. We also highlight the knowledge that being overweight is a bad prognosis factor due to inadequate dosage of medications for treatment, calculated from body weight, and the chronic inflammatory condition of obese patients [3, 32, 33].

The most worrying fact in obese patients might be related to the sarcopenia, characterized by a progressive and widespread loss of lean body mass, which is associated to a worsening of the functional status and quality of life and death [34]. A Canadian cohort that evaluated the sarcopenic obesity and its clinical implications in patients with solid tumors, found a prevalence of 15 % of sarcopenia in obese subjects (OR 4.2; 95 % CI: 2.4 – 7.2) [35].

Although it is well established in the scientific community that the types of cancer and treatment are predictive factors for malnutrition, in this study they did not remain in the final model. Such fact occurred probably due to the small sample for this analysis, because there were considered different types of response of these two variables [3, 6, 7]. It is important to consider that factors such as comorbidities, antibiotic therapy and diagnostic time can also be associated with the outcome, however, they were not collected and therefore are considered limiting factors of our research. It is also highlighted that the stage of the disease was not included on statistical analysis because the stage of tumors of different sites is distinct.

According to the extensive search performed in the main electronic databases, it was not found any other study in the northeast region of the country with a similar design, exclusively with hospitalized cancer patients, including palliative care, and using the PG-SGA. However, longitudinal studies that include larger numbers of patients to better determine the results found in our research are still needed.

## Conclusion

The data presented in this study showed the high prevalence of malnutrition in hospitalized cancer patients. The factors independently associated to this nutritional disorder were smokers/ex-smokers, socio-economic classes C, D and E, PS ≥2 and age ≥60 years. Thus, the simple perception of these factors can alert health professionals about the risk of nutritional depletion and the need for differentiated nutritional intervention.

## Availability of supporting data

The dataset that supports the results of this paper is included in the manuscript and its additional files.

## Abbreviations

% WL: Weight loss percentage
ABEP: Brazilian Association of Research Companies
ECOG: Eastern Cooperative Oncology Group
HW: Habitual weight
IMIP: Institute of Integrative Medicine Prof. Fernando Figueira
PAHO: Pan American Health Organization
pBMI: Previous body mass index
PG-SGA: Patient-generated subjective global assessment
PS: Performance status
SGA: Subjective global assessment
WHO: World Health Organization
